# Spatiotemporal imaging of complexity

**DOI:** 10.3389/fncom.2012.00101

**Published:** 2013-01-24

**Authors:** Stephen E. Robinson, Arnold J. Mandell, Richard Coppola

**Affiliations:** ^1^MEG Core Facility, National Institute of Mental Health, National Institutes of HealthBethesda, MD, USA; ^2^Department of Psychiatry, University CaliforniaSan Diego, CA, USA

**Keywords:** magnetoencephalography, neuroscience, cognitive, beamformer, complexity, nonlinear, turbulence, mixing

## Abstract

What are the functional neuroimaging measurements required for more fully characterizing the events and locations of neocortical activity? A prime assumption has been that modulation of cortical activity will inevitably be reflected in changes in energy utilization (for the most part) changes of glucose and oxygen consumption. Are such a measures complete and sufficient? More direct measures of cortical electrophysiological activity show event or task-related modulation of amplitude or band-limited oscillatory power. Using magnetoencephalography (MEG), these measures have been shown to correlate well with energy utilization sensitive BOLD fMRI. In this paper, we explore the existence of state changes in electrophysiological cortical activity that can occur independently of changes in averaged amplitude, source power or indices of metabolic rates. In addition, we demonstrate that such state changes can be described by applying a new measure of complexity, rank vector entropy (RVE), to source waveform estimates from beamformer-processed MEG. RVE is a non-parametric symbolic dynamic informational entropy measure that accommodates the wide dynamic range of measured brain signals while resolving its temporal variations. By representing the measurements by their rank values, RVE overcomes the problem of defining embedding space partitions without resorting to signal compression. This renders RVE-independent of absolute signal amplitude. In addition, this approach is robust, being relatively free of tunable parameters. We present examples of task-free and task-dependent MEG demonstrating that RVE provides new information by uncovering hidden dynamical structure in the apparent turbulent (or chaotic) dynamics of spontaneous cortical activity.

## Introduction

### Entropy and complexity

The term “entropy” is commonly defined as a measure of the order or disorder in a physical system. In the context of time-varying electrophysiological brain signals we use the term “complexity” instead of “entropy” in order to emphasize the temporal fluctuations of information rate rather than the total information of that signal. Signals having low complexity include synchronous events and oscillations. Signals with high complexity appear more chaotic and correspond to a higher information rate.

The topographic characteristics of spatiotemporal fluctuations in cortical electrophysiological activity are analogous to those of non-laminar, turbulent flow as visualized by optical imaging using voltage sensitive dyes (Cohen et al., 1978). On a macroscopic scale, the activity of individual neurons is hidden from external non-invasive measures such as magnetoencephalography (MEG) (Cohen, 1968) or electroencephalography (EEG) (Berger, 1969), the ensemble behavior of the underlying cortical neural network exhibits complex emergent and traveling fronts of excitation and inhibition that are supported by both short-range inter-neuron connections and “small world” longer-range connections. There have been a variety of experimental approaches that have been developed to characterize the dynamics of turbulent fluid flow ranging from visualization of waves and eddies through the use of fluorescent dyes (Busse and Clever, 1979) and monitoring heat flux using cryogenic techniques (Swinney and Gollub, 1981) to laser Doppler techniques that allow measurement of local field velocities without perturbing the field significantly (Gollub and Steinman, 1981). The spatiotemporal patterns observed with changing parameter values include transitions from laminar (linear) flow via a Hopf bifurcation to periodic oscillations, followed by two or more simultaneous irrationally related periodic flows and finally the aperiodic oscillations of turbulent (chaotic) dynamics (Ruelle and Takens, 1971). The latter state is characterized by positive entropy generation (Eckmann and Ruelle, 1985) similar to that observed in the MEG record (Mandell et al., 2011a,b; Robinson et al., 2012).

Brain activity is most commonly modeled by narrow-band oscillatory regions that are coupled to one another via networks. Chaos theory is often used as a model to describe complex biological measurements for which linear theory is incomplete. The criteria for chaos modeling include sensitivity to initial conditions and topological mixing. Both of these conditions are satisfied by local measures of ongoing brain activity. This naturally leads to combining a measurement of local cortical signals such as a beamformer estimate derived from MEG to a sensitive and robust measure of the broad bandwidth non-linear properties of those signals such as RVE.

The lack of suitable existing complexity measures for MEG is our motivation for developing rank vector entropy. First, we require a method for observing the spatiotemporal structure of cortical non-linear dynamics. Retaining temporal resolution allows for the study of the entropy change time-course that is needed to characterize event and task-related brain activity. The focus of current MEG complexity measures has been long-term properties having no temporal resolution such as Lempel-Ziv complexity (Fernandez et al., 2011), transfer complexity (Vakorin et al., 2010), and comparisons of multiple entropy/complexity measures (Bruna et al., 2012). Although there are sliding block methods for observing temporal changes in entropy (Adler and Marcus, 1979), this is computationally inefficient when applied to thousands of voxels for functional brain imaging. The estimated source time-series from beamformed MEG is efficiently transformed into an entropy time-series by RVE.

The *rank vector entropy* (RVE) algorithm is a non-parametric partial analog to metric (Kolmogorov) entropy (ME) (Kolmogorov, 1958; Crutchfield and Feldman, 2003). Both methods estimate the entropy of a one-dimensional series of measurements (e.g., a neurophysiological signal) computed on probability distributions of short sequence “states” encountered in the time series. A unique property of the RVE as a metric entropy lies in its initial encoding of the time series using the topological property of sequence order (Cornfield et al., 1982; Bandt and Pompe, 2002). In the more conventional ME, a short sequence of lagged measurement values from a one-dimensional time-series are mapped into an *N*-dimensional phase space (Ott, 1993). This embedding space is then partitioned into *N*-dimensional hypercubes. Each partition represents a state that the signal can manifest in its trajectory through *N*-dimensional space. The metric entropy of the signal is a measure on the probability distribution of trajectories passing through possible partitioned spaces. The number of partitions in ME can be arbitrary and must be sufficient in number, and with sufficient continuity to accommodate the dynamic range of the signal. In some implementations, dynamic range compression of the measurements is required in order to limit the number of partitions to a manageable number. The classical criteria of a “generating partition”—no more than one entry per partitioned space (Eckmann and Ruelle, 1985) is impractical in the context of real biological data. In contrast, in the RVE algorithm the measurement values of a short sequence of samples extracted from the entire time series are converted to their rank ordered values. The number of states, as partitions of the one-dimensional sliding window is quantized according to the number of elements in the sequence of measurements. Each sequence, with the topological property of relative “nearness,” is referred to as a “rank vector” which can also be thought of as a rank ordered one-dimensional embedding space. The RVE algorithm ignores the absolute signal amplitude in favor of its relative amplitude within the span of each sample window. A metric entropy is then computed on the probability distribution of the finite set of rank sequences. It will be shown that RVE is relatively free of arbitrarily tunable parameters (aside from selection of signal bandwidth, window length, and a decay time constant). The more conventional ME requires the investigator to use a variety of schemes, many involving the Whitney embedding theorem (Milnor, 1997; Temin, 1997), to determine the number of dimensions and lags for the embedding space and the size and number of partitions in embedding space.

We measure the spatiotemporal complexity of brain activity by applying the RVE analysis to sensor mediated brain signals. This could be accomplished by analyzing the signals from individual sensors (Vakorin et al., 2010; Gomez et al., 2011), pairs of sensors (Mandell et al., 2011a,b), or source estimates from MEG using a scalar LCMV beamformer (Robinson and Black, 1990; Robinson and Rose, 1993; Van Veen et al., 1997; Vrba and Robinson, 2002). The latter completes the analogy to characterization of time-series of turbulent chaotic fluid flow, sampled at multiple spatial points within the flow.

### Magnetoencephalography

Spontaneous MEG signals are on the order of 10^−13^ Tesla peak-to-peak. Despite its small signal strength, MEG has been made practical by larger DC-SQUID based sensor arrays covering the entire head in combination with excellent rejection of environmental magnetic interference (Fife et al., 2002). The application of RVE to MEG signals is a natural one. The magnetoencephalogram is the magnetic counterpart of the electroencephalogram. MEG is a measure of the magnetic field arising from primary (impressed) neural currents, whereas EEG measures the electrical potentials on the scalp that arise from the volume currents and is therefore dependent on tissue conductivity and its boundaries (Plonsey, 1981). The major contributor to the observed primary currents is the potential difference between the dendritic tree and soma of neocortical pyramidal neurons. Because MEG is less dependent on tissue conductivity its measurements can be modeled using simple analytic solutions (Sarvas, 1987). The accuracy of such analytic solutions for MEG enables the use of beamformers (detailed in section “Scalar LCMV Beamformer”) to estimate the source time series for any coordinate within the brain (Robinson, 1989). Application of the RVE transform (section “Rank Vector Entropy”) to any source time series yields a corresponding complexity time series of that activity. The relationship of spatiotemporal RVE to sensorimotor events and cognitive tasks can then be determined by signal averaging or by comparison of the RVE signals that have been parsed into active and control state time segments.

### Example MEG studies

We have selected four different examples of MEG studies to explore the properties of spatiotemporal RVE analysis. These datasets were selected from an archive of normal control studies. The task-free (resting) MEG dataset was chosen to compare the spatial distribution and timing relationships of RVE complexity to power in a 4–150 Hz bandpass. The P300 study, also referred to as the mismatch negativity (MMN), demonstrates the properties of evoked signals to frequent and deviant auditory stimuli. In the EEG, comparison of the frequent to deviant tone signal averages is characterized by a slow wave after about 250 ms (Naatanen et al., 1978). The same protocol is also referred to as a P_300_ study when a response is required for the deviant tones (Donchin, 1981). The working memory (n-back) study is used to compare broad-band RVE with power in an effortful short-term memory task (Kirchner, 1958). Lastly, we present a MEG study of self-paced voluntary finger movement to compare complexity with SCP. Self-paced voluntary movement is preceded by a slow “readiness” potential (Bereitschaftspotential) (Kornhuber and Deecke, 1964) and a corresponding “readiness” field (Bereitschaftsfield) (Deecke et al., 1982).

## Methods

### Rank vector entropy

The RVE algorithm can be described as follow: first, consider a one-dimensional discretely sampled time series of length *K* samples: **X** = {*x*_1_, *x*_2_, …, *x*_*K*_}. Let the sample rate and low-pass “corner” frequency of **X** be denoted by *f*_*S*_ and *f*_*C*_, respectively. For any given low-pass frequency, **X** is completely determined at 2*f*_*C*_ samples per second. It is unnecessary for the rank vector to represent every sample, sequentially. It is sufficient to define the lag ξ required to avoid oversampling as:

(1) $$\xi =\frac{f_{S}}{2f_{C}}.$$

Thus, for any specified low-pass cutoff and sample rate we need only process every ξth sample (ξ is rounded up to the nearest integer). Note the analogy of ξ to the sample lags that are used to define the dimensionality of the embedding space in conventional ME computations (Eckmann and Ruelle, 1985; Crutchfield and Feldman, 2003). The selection of lags in ME is usually based upon the mixing length reflected in the decay of the autocorrelation function (Walters, 1982). In this way it is biased in favor of the dominant signal and its ordering. These methods characteristically assume that the signal is stationary (which it is not) and it may also have the unfortunate side effect of aliasing information. Furthermore, it appears that with real biological data, some investigators have been occasionally arbitrary in their selection of lags (evaluating several different lags so as to obtain results more consistent with their expectations). In the RVE algorithm, the lags are rigorously defined by sample rate and low-pass corner frequency, thus not justifying any further modification. If the frequency band of interest is below the data acquisition bandwidth, the investigator can set the low-pass frequency accordingly, from which the lags are again automatically determined.

Let us select a sub-window of *W* samples (at integer ξ intervals) from **X**, with the window beginning with the *k*th sample: **W**_*k*_ = [*x*_*k*_, *x*_*k* + ξ_, …, *x*_*k* + (*W* − 1)ξ_]. The measured signal for each lagged sample within sub-window **W**_*k*_ is initially converted to its integer rank values, forming a rank vector **R**_*k*_ of length *W*: **R**_*k*_ = [*rank*_1_, *rank*_2_, …, *rank*_*W*_]. For a window of length *W* there are *W*! (factorial) unique rank vectors (i.e., vectors of length *W* for which the ordered rank values do not repeat). Let there be a table **S** to map rank vector sequences to symbols, where the symbol value is obtained from a “look up” table indexed for matching rank vectors. For example, let us consider a window length of *W* = 5 for which there are 5! = 120 unique rank vectors from which we derive 120 state symbols, *s*_*n*_. Counting and normalizing the number of occurrences of each unique symbol can then generate a probability histogram. Counting these symbols in RVE is analogous to counting visits of the signal to the higher dimensional hypercube partitions in ME (Ott, 1993). For this example, we use *W* = 5, *f*_*S*_ = 600 Hz, *f*_*C*_ = 100 Hz, and ξ = 3. For example, let the measured values of *x*_*k*_ through *x*_*k* + 4ξ_ (i.e., **W**_*k*_) be (4.07, −3.12, 3.95, 8.51, −1.21). Its rank vector and symbol value are (2, 5, 3, 1, 4) and 45, respectively (a symbol value of 45 indicates its place in an ascending numerical order of rank vectors).

As the sub-window of *W* samples is advanced through **X** one sample at a time, a new rank vector R_*k*_ and new symbolic representation *s*_*n*_ is generated. The frequency of occurrence of each of these symbols is accumulated in a corresponding state histogram: **F**_*k*_ = [*f*_1_ (*k*), *f*_2_ (*k*), …, *f*_*W*!_ (*k*)]. The resulting histogram contains the cumulative counts of each rank vector (state). Since our primary interest is in observing the time-dependent, event-related changes in the rank vector informational entropy, it is necessary to avoid the reduction in relative temporal sensitivity by saturation. We prevent this by introducing a time-constant determining the rate of decay of histogram counts with time. The integrator decay rate τ (1/*e* time) is required in order to measure the fluctuations in entropy over time. The amplitude of the entropy fluctuations depends on the decay rate (longer τ yields smaller peak-to-peak fluctuations). The relative rather than the absolute changes in entropy are of interest for event or task-related functional imaging (including ICA or resting state MEG). Provided that τ is longer than the time required to completely fill all states, the entropy waveform will be independent of τ—except for its amplitude. As a practical matter, we select a time constant such that the 1/*e* time (in samples) corresponds to three times the number of states. This is implemented by defining constant α:

(2) $$\alpha =e^{-1/\left(\tau f_{S}\right)}\text{​},$$

in which τ is the time for the counts to decay to 1/*e* of their initial values, such that for each time step:

(3) $$F_{k}=\alpha F_{k-1}\text{​}.$$

This constitutes what is termed a “leaky integrator.” The histogram count corresponding to the current state symbol is the incremented by one. Based upon the revised count frequencies, there will be a corresponding set of probabilities for each state:

(4) $$P_{k}=\left[p_{1}\left(k\right),\;p_{2}\left(k\right),\;\ldots ,\;p_{W!}\left(k\right)\right].$$

If the cumulative entropy over all samples of **X** is required, then α = 1. Otherwise, the entropy is estimated as time-dependent. Finally, for each step *k*, we compute the Shannon entropy (Shannon, 1963) over all state probabilities greater than zero, as normalized by its maximum value, constraining entropy to the range 0–1:

(5) $$h\left(k\right)=\frac{1}{\log_{2}W!}\sum_{n=1}^{W!}-p_{n}\left(k\right)\log_{2}p_{n}\left(k\right).$$

The steps in the algorithmic procedure for computing the time series of RVEs are as follows:

1. Initialize the state count histogram **F** (set all *f*_*n*_ values to 1.0).
2. For each sample index *k* in time series **X**, generate a length *W* rank vector **R**_*k*_ with lags of ξ samples.
3. Look up state symbol index *n* corresponding to **R**_*k*_.
4. Multiply all elements of histogram **F** by α, thus allowing the count histories to decay (Equation 3).
5. Increment the corresponding histogram count *f*_*n*_ by one (where *n* is the index in **S** corresponding to **R**_*k*_).
6. Compute the probabilities of each state from the histogram of counts (Equation 4).
7. Compute the RVE metric entropy for this time step (Equation 5).
8. Advance sample index by one sample and continue to repeat steps 2 through 7 and in this way generating a new entropy value for each cycle of the algorithmic process.

### Scalar LCMV beamformer

Synthetic aperture magnetometry (SAM) is a scalar linearly constrained minimum variance (LCMV) beamformer estimating source activity from MEG signals for specified coordinates in the brain. The mathematics of the beamformer procedure can be traced to the minimum variance estimator first described by Gauss (1823). SAM minimizes the variance (power) of all correlated signals observed by an array of SQUID sensors, subject to a unity gain constraint for a specified coordinate. As such, the action of the SAM may be regarded as spatially selective noise reduction, where noise is defined as unwanted environmental or biological magnetic signals.

The computational procedure has been described in detail elsewhere but can be summarized briefly, as follows: consider measured MEG from an array of sensors. Let the signal space vector at time sample *k* be denoted by **M**(*k*). Given a sufficient number of time samples, we construct a source estimate ${\hat{S}}_{r}$ for coordinate **r** as the weighted sum MEG measurements.

(6) $${\hat{S}}_{r}\left(k\right)=W_{r}^{T}M\left(k\right).$$

To compute the weights using the method of Gauss, we use the quadratic form:

(7) $${\left[{\hat{S}}_{r}\left(k\right)=W_{r}^{T}M\left(k\right)\right]}^{2},$$

for which, after integrating over time, we obtain:

(8) $${\hat{S}}_{r}^{2}=W_{r}^{T}CW_{\text{r}},$$

where **C** is the covariance matrix computed over the integration time:

(9) $$C=\langle MM^{T}\rangle ,$$

where <.> denotes the expectation value. We solve for **W** by introducing a constraint such that ${\hat{S}}_{r}^{2}$ (power or variance) is minimized subject to unit gain for a specified coordinate. One such constraint is:

(10) $$W_{r}^{T}B_{r}=1,$$

where **B_r_** is the *a priori* forward solution for the field observed by an array of *M* sensors generated by a current dipole source located at **r**. That is:

(11) $$B_{r}=\left[\begin{array}{c} b_{1}\left(r\right)\\ b_{2}\left(r\right)\\ \vdots \\ b_{M}\left(r\right) \end{array}\right].$$

Suitable methods for computing forward solutions include the current dipole in a homogeneously conducting sphere model (Grynszpan and Geselowitz, 1973; Sarvas, 1987; Hari et al., 1988), multiple local spheres fitted to the surface of the head (Huang et al., 1999), boundary element methods (De Munck, 1992) or perturbative solutions derived from the spherical harmonic expansion of the conductive boundary (Nolte et al., 2004). However, to compute **B**_**r**_ we must first estimate the dipole orientation. We do this by finding the dipole orientation yielding the highest signal-to-noise ratio (SNR) at location **r**. We define **L**_**r**_, the matrix of forward solutions for dipoles oriented in the three cardinal directions as:

(12) $$L_{r}=\left[\begin{array}{ccc} b_{11} & b_{12} & b_{13}\\ b_{21} & b_{22} & b_{23}\\ \vdots & \vdots & \vdots \\ b_{M1} & b_{M2} & b_{M3} \end{array}\right].$$

We also define a diagonal matrix Σ of uncorrelated sensor instrumental noise power σ^2^_m_:

(13) $$\Sigma =\left[\begin{array}{cccc} \sigma_{1}^{2} & & & 0\\ & \sigma_{2}^{2} & & \\ & & \ddots & \\ 0 & & & \sigma_{M}^{2} \end{array}\right]≅{\bar{\sigma }}^{2}I.$$

The source power **S** in each of three cardinal directions is given by:

(14) $$S_{r}^{2}={\left[L_{r}^{T}C^{-1}L_{r}\right]}^{-1},$$

and the noise power **N** by:

(15) $$N_{r}^{2}={\left[\Sigma \;L_{r}^{T}C^{-2}L_{r}\right]}^{-1}.$$

The SNR is given by a generalized eigensystem of these two 3 × 3 matrices (Sekihara and Nagarajan, 2008). By assuming that the SQUID noise is nearly equal in all sensors, we can neglect **Σ**, as it represents a scalar $\left({\bar{\sigma }}^{2}I\right)$ that will not affect determination of the moment vector. The dipole orientation maximizing SNR is given by the eigenvector **e**_*max*_ corresponding to the maximum eigenvalue λ_*max*_ of the generalized eigensystem:

(16) $$L_{r}^{T}C^{-2}L_{r}e_{k}=\lambda_{k}L_{r}^{T}C^{-1}L_{r}e_{k}.$$

The forward solution for the dipole vector for the highest SNR is therefore:

(17) $$B_{r}=L_{r}e_{\max}.$$

Solving for the optimum scalar LCMV beamformer weights using Lagrange multipliers results in:

(18) $$W_{r}=\frac{C^{-1}B_{r}}{B_{r}^{T}C^{-1}B_{r}}.$$

Substituting **W_r_** into Equation (6) yields an estimate of the source time series.

### Application of RVE to MEG data

#### Subjects

MEG data from four healthy normal control subjects, one per example study, were randomly selected from a larger group of NIMH study subjects of both genders, mean age 27.6 years. All subjects gave written informed consent according to protocols approved by the NIH CNS Institutional Review Board.

#### Data acquisition

MEG data were acquired using a 275-channel whole head MEG (CTF Systems, Inc.) housed within a three layer magnetically shielded room (Vacuumschmelze AK-3). Three head localization coils were affixed to subjects at the nasion, right preauricular, and left preauricular points. The acquisition software energizes these coils with sinusoidal currents at three different frequencies before and after data acquisition in order to localize and establish each subject's head position relative to the MEG sensors. Data were sampled continuously, without breaking it into epochs or trials at 600 Hz in a bandpass of DC to 150 Hz, with the subjects in seated position. Stimulus and response trigger markers for each study were recorded with the data. Synthetic 3rd-gradient mode was used during data acquisition to obtain further reduction in magnetic noise (Vrba and Robinson, 2002). Raw data were archived on disk for subsequent analysis.

RVE analysis parameters for all studies were fixed at a 4–150 Hz bandpass, *W* = 5 (120 states), lags every two samples, and an integrator decay time constant of 0.6 s.

All subjects were given a T1-weighted volumetric MRI. Radiological markers were affixed to the identical fiducial points as were used for the head localization coils used during MEG acquisition. Markers were used to transform the MRI to the MEG head frame for subsequent processing, including segmentation of the cortical boundary, and coregistration of functional and anatomical data.

#### Data analysis

Analyses were applied to the unaveraged continuous data. We use the single-layer realistic head model (Nolte et al., 2001) to compute the forward solutions. The scalar beamformer processing includes the steps:

1. Coregister a T1-weighted MRI to the MEG frame and segment the MRI to extract subject's brain hull, using AFNI software (Cox, 1996).
2. Compute the outward-pointing normal vectors for the brain hull.
3. Estimate the MEG measurement covariance matrix for the required time segments and frequency bandpass (Equation 9).
4. Compute points (voxel coordinates) on a regular three-dimensional grid at 5 mm intervals within the head.
5. For each voxel within the hull boundary:
  - Compute the lead-field matrix (Equation 12) using the brain hull as a single-layer realistic head model (Nolte et al., 2001).
  - Compute the beamformer coefficients (Equation 18).
  - Estimate the source time series (Equation 6).

The RVE voxel time-series is computed for a 4–150 Hz bandpass (Equation 1–5). No additional filtering or smoothing is required as the RVE time-series is inherently smooth. Source power time-series are computed from the smoothed envelope of a Hilbert transform following bandpass filtering of the MEG data. The Hilbert envelope was smoothed using lowpass filter corresponding to the lowpass corner frequency of each selected bandpass.

The fluctuations in RVE are relatively small (on the order of 5–15% for α = 0.6 s). RVE deviation relative to its statistical mode is used for analysis of the task-free data. For the remaining studies, we compute the Student's T-value for each latency in the RVE time, relative to a selected baseline. Static (3D) and spatiotemporal images (3D + time) were assembled and displayed using AFNI software (Cox, 1996).

#### Task-free (resting) protocol

Two hundred and forty seconds of task-free (“resting') MEG data were recorded from a normal subject (eyes opened), using data acquisition procedures outlined in section “Data Acquisition.” Beamformer weights were computed as above for the entire duration at 5 mm voxel intervals on a three-dimensional grid occupying the entire head. The envelope of the source power *S*^2^ and the RVE were then computed for each voxel and the results mapped as three dimensions plus time images at 50 ms intervals. Because RVE is a measure on temporally hierarchical brain signals, it is studied using a broad bandwidth of 4–150 Hz. In this way we avoid the potential reduction in complexity that would accompany our narrowing the bandwidth of observation. In comparing RVE, with the simultaneously studied envelopes of band width power, the latter was computed using a smoothed Hilbert transform for a sequence of bandwidths that included: 4–150 Hz, 4–8 Hz (theta), 8–13 Hz (alpha), 15–30 Hz (beta), 35–70 Hz (low gamma), and 70–150 Hz (high gamma). The RVE time series is inherently smooth and required no additional filtering.

#### Auditory P300 (mismatched negativity) protocol

The auditory P300 protocol consisted of random presentation of 200 frequent (1.0 kHz) and 50 rare (1.5 kHz) 50 ms duration tone bursts delivered binaurally via non-magnetic earphones (Etymotic), with a 1.0–1.5 s pseudo-random interstimulus interval (ISI). Subjects were instructed to respond to the rare tones by pressing a response button. MEG data were acquired as noted in section “Data Acquisition” and stored to disk together with trigger markers for identifying the onset of the rare and frequent tone bursts, along with the button response.

Beamformer weights were computed from the *unaveraged* MEG data for each of three conditions: (1) both frequent and rare tones, (2) frequent tones, and (3) rare tones. The weights were computed in a 4–150 Hz bandpass. Three-dimensional images of RVE were then computed at 5 ms intervals over a time window from −0.2 to +0.8 s relative to the markers for each of the three conditions. Image maxima and minima coordinates were determined and additional beamformer weights computed for a DC to 100 Hz bandpass. These weights were then applied to the averaged RVE signal of the three conditions in order to show the time course of the entropy waveforms. Note that we compute the average of the entropy and *not* the entropy of the averaged signal. The RVE functional images and time series are displayed as Student's T-values for each voxel, relative to the selected pre-stimulus baseline.

#### Working memory (N-back) protocol

The numerals 1 through 4 were randomly presented to the subject at 1.4-s intervals in 18 blocks of 11 trials each, using a DLP projector. Preceding each block the subject received instructions how to respond to via four numbered buttons, that corresponds to the numbers 1 through 4. For 0-back blocks, the subject simply pressed the button corresponding to the number presented. For 1 and 2-back conditions, the subject pressed the button corresponding to the numbers that were presented one or two trials back, respectively. MEG was recorded, along with trigger markers indicating the task (i.e., respond to 0, 1, or 2-back), which number was displayed and which response button was depressed, using the settings in section “Data acquisition.”

The RVE was applied to the continuous source time series of each voxel, resulting in an RVE time series. The RVE voxel time series was then parsed into 0.5-s segments (± 0.25 s relative to the button response) for the 0, 1, and 2-back conditions. The RVE was integrated over each segment. A Student's *T*-test was used to compare the difference in integrated RVE signal for pairs of each condition (i.e., 2 vs. 0-back, 2 vs. 1-back) for each individual voxel. The results were displayed as *p*-values using AFNI.

In a similar manner, we computed and displayed the comparisons for beta-band (14–30 Hz) power using the same conditions.

#### Self-paced voluntary movement protocol

Subjects performed self-paced button presses at intervals of at least 10 s while continuous MEG was recorded together with time markers for each button press, as outlined in section “Data acquisition.” No cues were given to the subjects as to when to press the button.

Scalar beamformer weights were computed for the entire unaveraged dataset in a bandpass of 4–150 Hz. Weights were then applied to the measured MEG data yielding a source estimate time series for each voxel. The voxel time series was then transformed to a RVE time series. The mean entropy value over the interval from −3.0 to −2.5 s prior to the button press was designated as a baseline for comparison of later entropy changes; the RVE time-series was averaged relative to the voluntary button press, at 5 ms intervals from −3.0 to 2.0 s. The RVE averaged response is displayed as its Student's T-value relative to the selected baseline. The voxel having the maximum relative RVE prior to the button press was also used to compute the averaged source strength time-series.

## Results

### Task-free (resting) protocol

**Figure 1:**
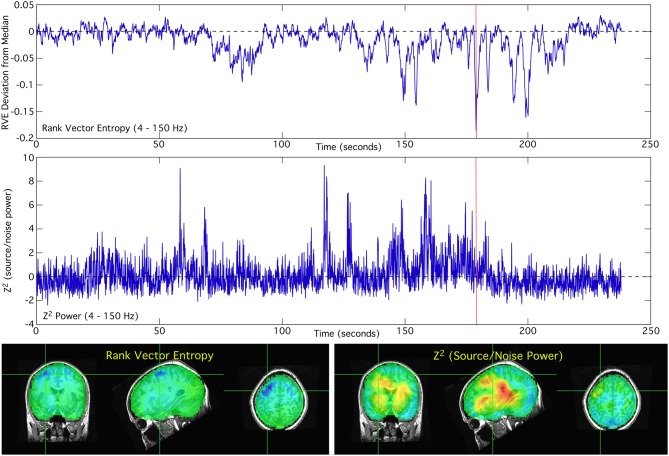
**Rank vector entropy and noise-normalized source power differed in both locations and dynamical patterns, i.e., in both time and space.** The time-courses of RVE and power for the 4–150 Hz bandwidth are shown for a single location in the brain, as indicated by the cross-hairs in the functional images. The functional images of RVE and power correspond to activity at the time indicated by the red cursor.

Comparison of the spatial and temporal patterns in the 4–150 Hz bandpass reveals very little apparent correlation between RVE and the Hilbert envelope of power. The fluctuations in entropy are noticeably slower than that of power. In many cortical locations the RVE shows transient decreases from its modal value; spontaneous entropy increases are not as prominent in the resting condition (Figure 1 top panel). Changes in power relative to its modal value (Figure 1 center panel) are much more rapid than those of the RVE. The spatial distribution of RVE and power also are seen to differ (Figure 1 bottom panel).

**Figure 2:**
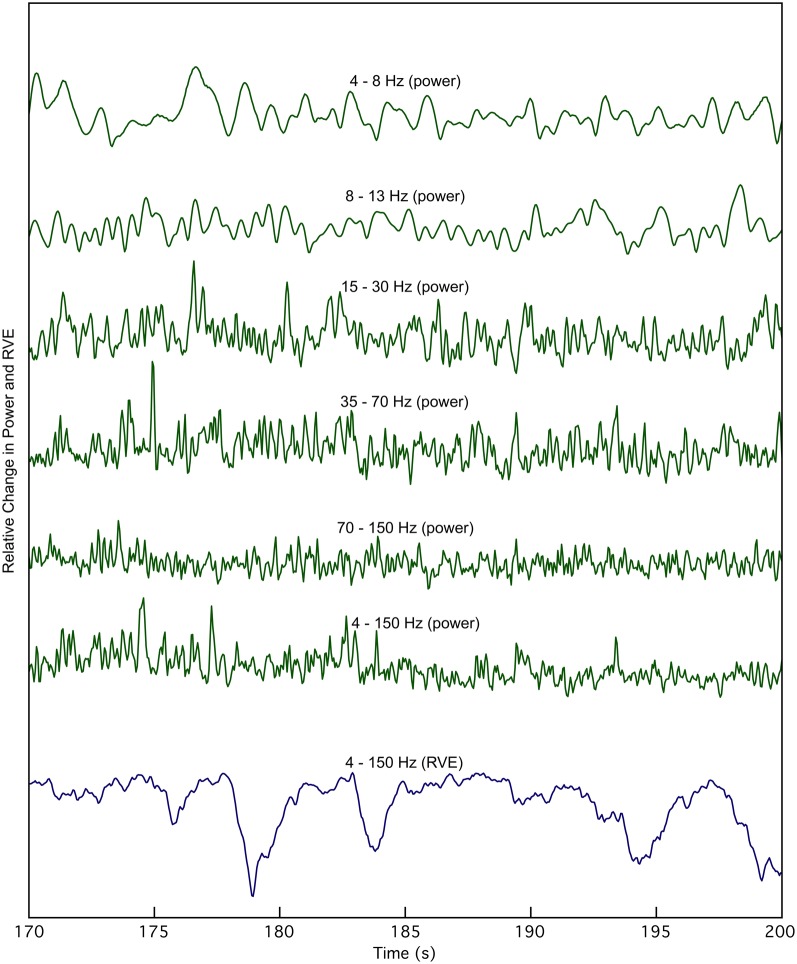
**The time course of rank vector entropy, *h*(*k*) (blue trace), is apparently uncorrelated with changes in the envelope of source power, in any bandpass.** The 4–150 Hz RVE is compared with source power at the same location in multiple bandwidths for a 30 s expanded view derived from the waveforms shown in Figure 1.

An expanded 30-s view of the time course of the smoothed Hilbert envelope of power and across multiple frequency bands shows no apparent correlation with RVE 4–150 Hz (Figure 2). Changes in entropy clearly show slower, longer wavelength features that are not evident in measures of power.

### Auditory P300 task

**Figure 3:**
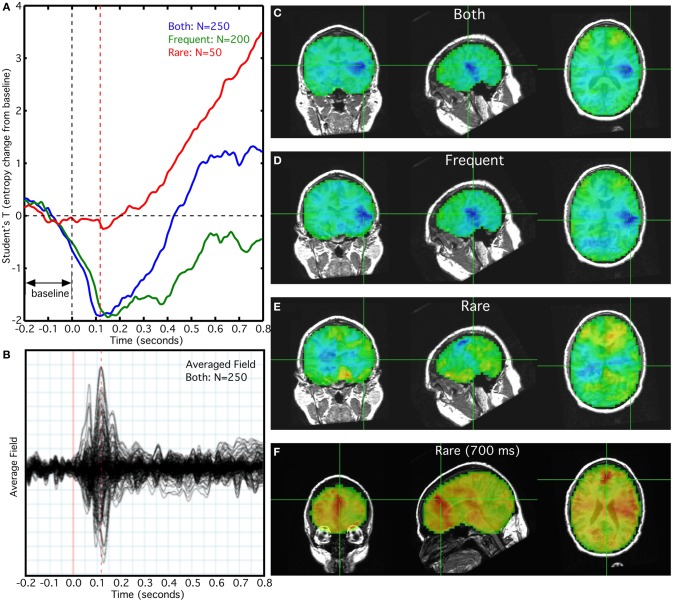
**Relationship of rank vector entropy to the auditory evoked response to rare and frequent tone bursts. (A)** Average of entropy time series for left primary auditory cortex; **(B)** average overlay of 275 MEG sensors; **(C)** RVE image of combined rare and frequent tones at 100 ms; **(D)** RVE image of frequent tones at 100 ms; **(E)** RVE image of rare tones at 100 ms; **(F)** RVE image of rare tones at 700 ms latency.

In this characteristic example, we observed a reduction in entropy coinciding with the N_100M_ peak (Figure 3B) for frequent and combined frequent and rare trials (Figure 3A). The reduction in entropy at 100 ms was much smaller for the rare tones, alone (Figure 3B). The reduction in entropy was maximal in the vicinity of the left Heschl's gyrus (Figures 3C,D,E), with a much smaller reduction in the right hemisphere. Although a flat baseline was seen in the pre-stimulus interval of −0.2 to 0.0 s in the averaged overlay of all sensors (Figure 3B), it is absent from the combined and frequent trial averages (Figure 3A). We also observed an increase in the entropy T-value after 100 ms for the rare tones (Figure 3A). At 700 ms this increase was maximal in anterior cingulate and left temporal cortex (Figure 3F). The increase does not appear in the sensor signal averaged overlay.

### Working memory task

**Figure 4:**
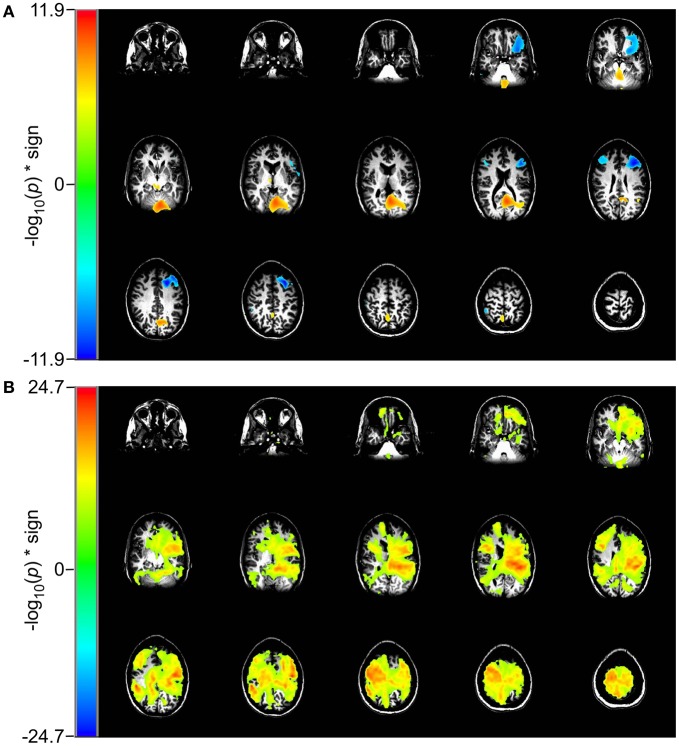
**Comparison of event-related changes in beta-band power and in rank vector entropy for 2 vs. 0-back working memory task centered on the response.** Images are univariate *p*-values for a Student's *T*-test, with a threshold of 10^−5^. **(A)** Event-related desynchronization of beta band power (14–30 Hz) appears bilaterally in dorsolateral prefrontal cortex and in left supraorbital prefrontal cortex. **(B)** RVE (4–150 Hz) shows widely distributed increases that overlap with regions of beta-band ERD.

In the 2 vs. 0-back comparison, we observe a reduction in beta-band power in the 2-back task relative to the 0-back task, ERD, in dorsolateral prefrontal cortex (DLPFC) and an increase in power, ERS, in inferior occipital cortex (Figure 4A). For the identical comparison, there is a significant increase in entropy (*p* < 10^−5^) in the 2-back condition that is widely distributed throughout the brain, with the largest changes in the left hemisphere (Figure 4B).

**Figure 5:**
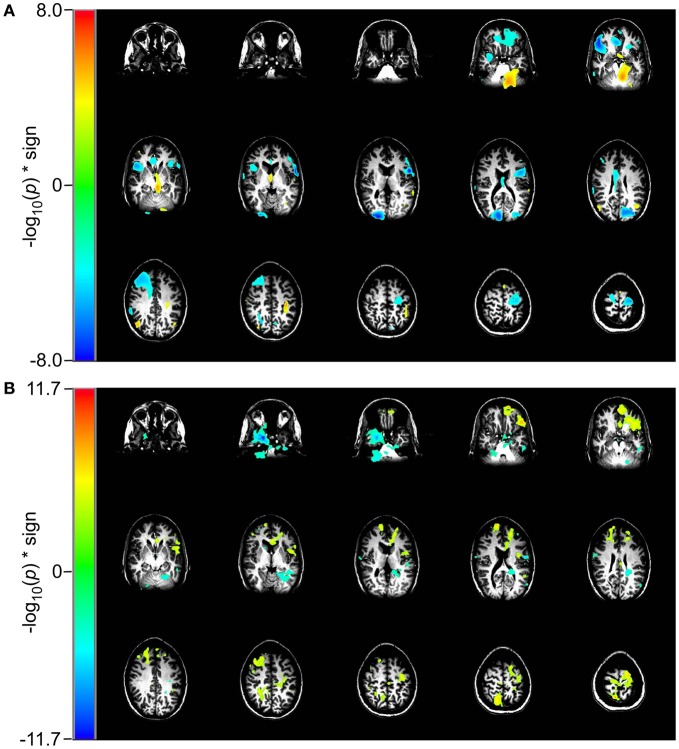
**Comparison of event-related changes in beta-band power and in entropy for 2 vs. 1-back working memory task centered on the response.** Images are univariate *p*-values for a Student's *T*-test, with a threshold of 10^−3^. **(A)** Event-related desynchronization of beta band power (14–30 Hz) and **(B)** RVE (4–150 Hz).

In the 2 vs. 1-back comparison, beta-band ERD is seen in DLPFC and bilaterally near the inferior temporal pole (Figure 5A). The same comparison for RVE shows an increase in entropy in anterior cingulate cortex and a decrease in the right inferior temporal pole (Figure 5B).

### Self-paced voluntary finger movement

**Figure 6:**
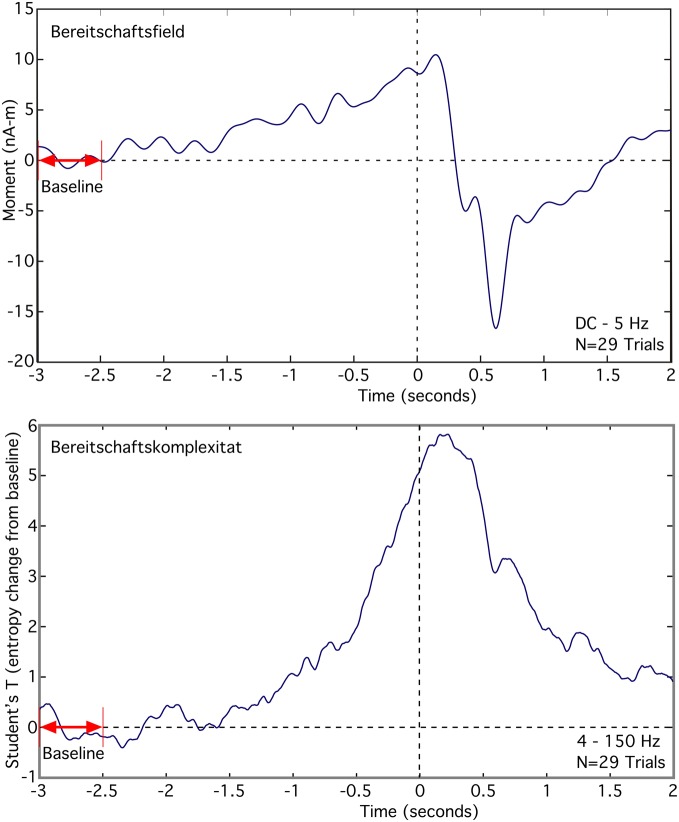
**Comparison of the averaged Bereitschaftsfield (DC to 5 Hz) to the averaged Bereitschaftskomplexität RVE (4–150 Hz) for a single voxel in premotor cortex contralateral to a self-paced voluntary finger movement (button press)**.

The Bereitschaftsfield (BF), average of 29 trials, appears as a slow low frequency rise in the source moment in premotor cortex that occurs over the time span from −2.5 s to 0 s, relative to the button press (Figure 6 top). We observed a peak dipole moment of about 10 nA -m relative to a mean baseline over the interval −3.0 to −2.5 s. Signal-to-noise ratio for this source is low, requiring a lowpass filter of 5 Hz in order to see the slow changes. By contrast, the averaged RVE (4–150 Hz), displayed as a T-value relative to baseline, has excellent signal-to-noise and spans −1.75 to 0 s (Figure 6 bottom). We refer to this wave as the “Bereitschaftskomplexität” (BK), to emphasize its relationship to the BF. Both the BF and BK waveforms are similar.

## Discussion

Our single most important finding is that the RVE measure of complexity adds new information about brain dynamics that was previously hidden within the apparent chaos of spontaneous cortical signals. Moreover, RVE, when combined with a scalar beamformer, reveals an underlying spatiotemporal complexity pattern that is modulated by stimuli and tasks. We have presented experimental evidence demonstrating that these patterns of complexity are decoupled from conventional measures of amplitude and oscillatory power. Thus, RVE reveals new information on the dynamics of brain activity.

Next, we will examine the experimental evidence regarding what directional changes in entropy signify. MEG measures such as band-limited oscillatory power or amplitude have been shown to be concordant with functional imaging by BOLD fMRI (Singh et al., 2002; Coppola et al., 2004). Induced changes in cortical activity, particularly for movement, are signaled by event-related desynchronization (ERD) of power (Taniguchi et al., 2000) and also by increases in the BOLD signal relative to a resting or control state. How does one interpret event-related changes in RVE? The answer is not straightforward. We show that a simple auditory stimulus induces a decrease in the RVE at a latency corresponding to the N_100_m and location corresponding to primary auditory cortex in the left hemisphere. Comparable MEG studies also show ERD in the beta and alpha bands at the latency and locations corresponding to the N_100_m, and fMRI shows a corresponding increase in the BOLD signal.

In our auditory P_300_ study the response to the rare tones elicited later increases in the RVE signal that persisted for over 1 s. The increase was seen broadly in anterior cingulate cortex and in left temporal cortex in a wide area centered on where the N_100_m response appeared. These late responses are not visible in the averaged overlay of sensors and in the event-related changes in power. We see that the absence of a stable baseline for the frequent tones is a consequence of the slow return to baseline of the entropy increase evoked by the rare tones. That is, the 1.0–1.5 s ISI is not sufficient for the induced RVE increase to return to its resting value.

These P_300_ results demonstrate that stimuli and tasks can induce either in increase or a decrease in entropy relative to its resting value. The RVE of task-free MEG is characterized by transient decreases in entropy from its modal value of ~0.92, lasting two or more seconds. Increases above the modal value are not as prominent and are more rapid—suggesting that bidirectional modulation of RVE reflects the parabolic character of the entropy function and/or its governance by at least two different mechanisms. The N_100_ evoked component of frequent tones corresponds to a transient decrease in the RVE signal that is much shorter than the observed transients in the task-free MEG recordings. The localization of decrease was centered on the left auditory cortex, but included perisylvian areas. The broad distribution of the induced entropy changes confirm that the auditory N_100_ component arises from multiple regions and not simply primary auditory cortex (Naatanen and Picton, 1987). Although the averaged evoked response to the rare tones showed only a small peak corresponding to the P_300_ response, there was a profound slow increase in the RVE signal starting at about 250 ms. This slow increase is concordant with the mismatch negativity signal that is observed in similar protocols involving frequent and deviant tones. It appears that synchronized cortical activity, such as that leading to an averaged evoked response component, induces a transient decrease in entropy in the corresponding regions. By contrast, the slow and prolonged rise in entropy induced by the rare tones suggests an increase in asynchronous cortical activity that signifies attentional mechanisms. It should be noted that the frequent tone average (Figure 3A) shows what appears to be activity where the pre-stimulus baseline should instead be flat. This is an artifact due to the long duration of the slow RVE component of the rare tones and the relatively short 1.5 s interstimulus interval.

RVE's relatively long time scales (in seconds) and the continuity and differentiability in its time-dependent changes suggests a relationship of this measure to the family of slow cortical potentials (SCPs), including the contingent negative variation (CNV) and the Bereitschaftsfield (Birbaumer et al., 1990). This is particularly evident when comparing BF and BK waveforms seen in Figure 6. The slow changes in entropy are only partially due to integration of symbolic state counts. The decay time constant incorporated into the integrator permits RVE to respond rapidly to changing complexity. The observed slow changes in RVE are significantly longer than the 0.6-s 1/*e* integration decay time constant used in these analyses. Thus, the slow shifts in entropy are not an artifact of the analysis but rather reflect cortical processes. Further studies will be needed to compare RVE with other SCP phenomena (e.g., the contingent negative variation paradigm and orienting responses).

The working memory task lends additional insight into how the RVE complexity measure is modulated by cognitive effort. The contrast for the 2-back to the 0-back condition for RVE (Figure 4) shows widespread and highly significant (*p* < 10^−5^) increases in entropy throughout the brain. The 2-back task engages working memory whereas the 0-back task does not. Although the RVE spatial distribution is not uniform, it suggests greater and more significant changes than does event-related beta-band power. These same conditions viewed as beta-band power show the expected focal changes in DLPFC. The difference between RVE and beta-band power images suggests that there vast tracts of cortex that have changed into an “up” state (i.e., activation of the thalamo-cortical attention circuit), without significant expenditure of energy. Is this arousal? Is it readiness? One might speculate that this widespread *increase* in entropy represents a decrease in the cortical excitability threshold—necessary for the efficient performance of the 2-back task. Such a mechanism would be concordant with our observations of other slow changes in RVE, such as that seen in the self-paced voluntary finger movement study and late P_300_ components for the rare tones. By contrast, the 2-back and 1-back conditions both engage working memory but with different levels of effort. For this comparison the RVE demonstrates a number of focal increases bilaterally in anterior cingulate gyri, supraorbital prefrontal cortex, and a focal decrease in the left inferior temporal pole (*p* < 10^−11^) as shown in Figure 5. Thus, the contrast for increasing memory workload can result in both increases and decreases in RVE. The RVE results are qualitatively different from the same contrasts in task-related beta-band power (Figures 4 and 5). Beta-band changes appear mainly as ERD in dorsolateral prefrontal and parietal cortex for both 2 vs. 0-back and 2 vs. 1-back. The *p*-values for these regions are smaller than those for the RVE. Overall, the working memory task suggests the higher cognitive load is associated with increases in RVE.

The results obtained from these MEG studies provide evidence that RVE—specifically increases in complexity—are measures of attention and intention. The neurophysiological basis of attentional arises from the non-specific projections from the centromedian thalamic nucleus to the neocortex (Steriade, 1995). Activation of these projections depolarizes the apical dendrites of neocortical pyramidal neurons resulting in a decrease in the excitability threshold. An increase in asynchronous firing rate of these neurons will be reflected by an increase in complexity, as seen in the RVE signal. It should also be noted that this same mechanism is responsible for the low frequency negative signal appearing in the scalp EEG—the so-called SCP (Birbaumer et al., 1990). Detection of the EEG SCP signals requires careful removal of motion artifacts, eye-blinks, and higher frequency signals and rhythms. By contrast the RVE signals corresponding to attention are readily measured in a broad bandwidth with little or no filtering. Thus RVE is a more sensitive measure of attentional mechanisms than is the SCP.

The complexity decrease associated with synchronous and evoked events such as the auditory N100 implies a *low information rate*. This calls to question the model that synchronous events represent transfer of information. Instead, we suggest a new model in which synchronous activity signifies degradation of the current cognitive context in a cortical region so that new information from sensory or associative sources can be incorporated into a new cognitive context. Exploration of this model is the subject of our ongoing research.

The RVE combines some properties of topological (relative nearness as non-numeric sequences) and metric (probability measure theoretic) entropies (Cornfield et al., 1982; Ornstein, 1989; Milnor, 1997). The log density of states (Equation 7) is a metric entropy. These states are a consequence of mapping topologically ordered sequences to vectorial states, then mapping these states into a symbolic dynamic topology, eventuating in the metric transformation of the density distribution of these symbolic states (Pollicott and Yuri, 1998).

The RVE is distinguished from ME in the order of operations. In ME, the states are defined by discrete partitions in the embedding space of *measured values* and the probabilities are derived from the number of counts in each *n*-dimensional partition. It can be difficult to define the boundaries of each partition—particularly when signals cover a wide dynamic range. In such cases, signal amplitude compression may be required. By contrast, RVE reduces the measurements to their rank values for each length *W* window. Each rank vector represents a state symbol (Adler et al., 1977) and partitions, as such, are no longer required; it only necessary to count the instances of each symbol. This step not only defines a finite number of available states but also renders the entropy measure-independent of absolute signal amplitude.

As a consequence of the integrator decay time constant, the mean of the entropy time series will be smaller than in the limit of α = 1 for which integration takes place over all samples. A similar decrement from the maximum entropy was also found in older methods (Walters, 1982). This decay time constant is required in order to maintain sensitivity to entropy changes over time. As expected, RVE approaches its limiting value as the decay constant is increased. At long decay constants fluctuations will be very small due to the accruing “memory” of the state counts. For MEG data sampled at 600 Hz and *W* = 5, we choose a 1/*e* time constant 0.6 s—corresponding to three times the number of states, in samples. This is more efficient than the sliding block methods for which entropy is computed for the number of samples in each block, and the blocks are advanced one sample at a time (Adler and Marcus, 1979). It should be mentioned here that altering the decay time constant changes the peak-to-peak amplitude of the entropy fluctuations, but not its waveform (thus the RVE measure)—provided the time constant is sufficiently long to allow the state counts to fully populate.

There is an inherent frequency bias in the RVE method. The duration of the sub-window from which the rank vector is obtained constitutes a highpass filter on the signal. To a measureable extent the contribution of the entropy of signals with wavelengths longer than this sub-window is reduced. This suggests future innovations of the RVE algorithm for the purpose of better conserving the range of broadband responses. For example, one could apply a 6 dB per octave increase in the measured signal for frequencies below the window cutoff to compensate for the decline in sensitivity.

The tunable parameters for RVE analysis are the rank vector length, signal lowpass frequency, and integration decay rate. We here note practical limitations of rank vector length. Since the number of possible states is exactly determined by the length of the rank vector, if *W* = 4, 5, and 6 there are 24, 120, and 720 possible states, respectively. As a compromise, *W* = 5 seems most attractive because it allows for a sufficient number of states to reflect the probabilities without too many or too few states to be impractical (i.e., to not fully represent the latent structure in the time-series). For example, when *W* = 3, there are only 6 allowable states and when *W* = 7 there are 5040 allowable states. The latter would require a very large number of samples to accurately reflect the probabilities of each state, while the former would provide very poor state resolution. The lags defining the phase space are determined by the signal lowpass frequency. Since signal complexity decreases as bandwidth is reduced, it defeats the purpose of complexity analysis to limit the bandwidth. Therefore, the low pass should reflect the information content of the measured signal. Lastly, the integrator decay rate is selected so as to allow the state count histogram to be well-populated (a sufficient number symbol counts for obtaining a good estimate of the entropy) and have a short lead-in time (i.e., the time required for the entropy to approach its asymptotic value). For 120 states, and a sample rate of 600 Hz, a 1/*e* time of 0.6 s is sufficient. Note that, as indicated above, the integrator decay does not lowpass filter the entropy waveform. It only affects the relative amplitude of the entropy fluctuations around the asymptotic value.

The normal cerebral cortex is never quiescent—even when not engaged in specific tasks. We have shown that stimuli and tasks modulate the regional entropy of cortical activity, inducing either increases or decreases in our RVE measure. The question posed initially was whether RVE provides new information on brain activity that is not obvious when observing only changes in source power. We conclude that RVE is a sensitive measure of brain signal complexity that complements other MEG functional imaging techniques including event and task-related changes in power.

### Conflict of interest statement

The authors declare that the research was conducted in the absence of any commercial or financial relationships that could be construed as a potential conflict of interest.
